# Immunogenomic analyses associate immunological alterations with mismatch repair defects in prostate cancer

**DOI:** 10.1172/JCI121924

**Published:** 2018-09-04

**Authors:** Daniel Nava Rodrigues, Pasquale Rescigno, David Liu, Wei Yuan, Suzanne Carreira, Maryou B. Lambros, George Seed, Joaquin Mateo, Ruth Riisnaes, Stephanie Mullane, Claire Margolis, Diana Miao, Susana Miranda, David Dolling, Matthew Clarke, Claudia Bertan, Mateus Crespo, Gunther Boysen, Ana Ferreira, Adam Sharp, Ines Figueiredo, Daniel Keliher, Saud Aldubayan, Kelly P. Burke, Semini Sumanasuriya, Mariane Sousa Fontes, Diletta Bianchini, Zafeiris Zafeiriou, Larissa Sena Teixeira Mendes, Kent Mouw, Michael T. Schweizer, Colin C. Pritchard, Stephen Salipante, Mary-Ellen Taplin, Himisha Beltran, Mark A. Rubin, Marcin Cieslik, Dan Robinson, Elizabeth Heath, Nikolaus Schultz, Joshua Armenia, Wassim Abida, Howard Scher, Christopher Lord, Alan D’Andrea, Charles L. Sawyers, Arul M. Chinnaiyan, Andrea Alimonti, Peter S. Nelson, Charles G. Drake, Eliezer M. Van Allen, Johann S. de Bono

**Affiliations:** 1 The Institute of Cancer Research, London, United Kingdom.; 2 The Royal Marsden, London, United Kingdom.; 3 Department of Clinical Medicine and Surgery, Department of Translational Medical Sciences, Azienda Ospedaliera Universitaria (AOU) Federico II, Naples, Italy.; 4 Department of Medical Oncology, Dana-Farber Cancer Institute, Boston, Massachusetts, USA.; 5 The Broad Institute, Cambridge, Massachusetts, USA.; 6 University of Washington, Seattle, Washington, USA.; 7 Fred Hutchinson Cancer Research Center, Seattle, Washington, USA.; 8 Weill Medical College of Cornell University, New York, New York, USA.; 9 University of Michigan Medical School, Ann Arbor, Michigan, USA.; 10 Karmanos Cancer Institute, Detroit, Michigan, USA.; 11 Memorial Sloan Kettering Cancer Center, New York, New York, USA.; 12 Institute of Oncology Research (IOR), Bellinzona and Faculty of Biomedical Sciences, Università della Svizzera Italiana, Bellinzona, Switzerland.; 13 Columbia University Medical Center, New York, New York, USA.

**Keywords:** Genetics, Oncology, Cancer immunotherapy, DNA repair, Prostate cancer

## Abstract

**BACKGROUND.** Understanding the integrated immunogenomic landscape of advanced prostate cancer (APC) could impact stratified treatment selection.

**METHODS.** Defective mismatch repair (dMMR) status was determined by either loss of mismatch repair protein expression on IHC or microsatellite instability (MSI) by PCR in 127 APC biopsies from 124 patients (Royal Marsden [RMH] cohort); MSI by targeted panel next-generation sequencing (MSINGS) was then evaluated in the same cohort and in 254 APC samples from the Stand Up To Cancer/Prostate Cancer Foundation (SU2C/PCF). Whole exome sequencing (WES) data from this latter cohort were analyzed for pathogenic MMR gene variants, mutational load, and mutational signatures. Transcriptomic data, available for 168 samples, was also performed.

**RESULTS.** Overall, 8.1% of patients in the RMH cohort had some evidence of dMMR, which associated with decreased overall survival. Higher MSINGS scores associated with dMMR, and these APCs were enriched for higher T cell infiltration and PD-L1 protein expression. Exome MSINGS scores strongly correlated with targeted panel MSINGS scores (**r** = 0.73, **P** < 0.0001), and higher MSINGS scores associated with dMMR mutational signatures in APC exomes. dMMR mutational signatures also associated with MMR gene mutations and increased immune cell, immune checkpoint, and T cell–associated transcripts. APC with dMMR mutational signatures overexpressed a variety of immune transcripts, including CD200R1, BTLA, PD-L1, PD-L2, ADORA2A, PIK3CG, and TIGIT.

**CONCLUSION.** These data could impact immune target selection, combination therapeutic strategy selection, and selection of predictive biomarkers for immunotherapy in APC.

**FUNDING.** We acknowledge funding support from Movember, Prostate Cancer UK, The Prostate Cancer Foundation, SU2C, and Cancer Research UK.

## Introduction

Understanding the relationship between tumor genomics and the immune response in advanced prostate cancer (APC) has acquired major therapeutic relevance with the advent of immunotherapy, especially after the failure of anti-CTLA4 (ipilimumab) in 2 large phase III trials in unselected patients (1, 2). Of paramount importance, recent studies indicate that mismatch repair–defective (dMMR) cancers may benefit from immune checkpoint–inhibiting therapies (3), regardless of tissue of origin. Initial clinical data suggest that 5%–12% of patients with metastatic castration-resistant prostate cancer (mCRPC) may benefit from immune checkpoint blockade (4, 5). A variable prevalence (12%–22%) of dMMR machinery has been reported in different APC studies, and this could be related to technical limitations of distinct assays available to detect these genomic aberrations (6, 7).

The mismatch repair (MMR) system is a post-replicative, high-fidelity, single-strand repair mechanism that recognizes and reverses DNA base mismatches and insertion/deletion (indel) loops; compromised MMR results in microsatellite instability and a hypermutator phenotype that has been associated with chemotherapy resistance but immunotherapy sensitivity (8). Immunotherapy-sensitive cancers such as melanomas tend to harbor high mutational loads (9, 10), which have been positively correlated with neoantigen burden (11). Conversely, mCRPCs on average have lower detectable mutation loads of approximately 4 mutations/megabase (12).

Multiple approaches have been developed to leverage molecular tumor profiling data toward that end, including mutational signatures associated with dMMR (10) and evaluation of microsatellite instability (MSI) using next-generation sequencing (NGS) (MSI-NGS) (13). Here we conducted an integrated characterization of clinical, pathologic, genomic, and immunologic features of 2 large APC cohorts. By analyzing a cohort of 124 Royal Marsden patients with non-indolent prostate cancer, with 85 hormone-naive prostate cancer (HNPC) and 127 mCRPC tissue samples available, and 254 mCRPC tumors from the Stand Up To Cancer/Prostate Cancer Foundation (SU2C/PCF) database, we aimed to characterize: (a) differences in clinical behavior between dMMR and non-dMMR prostate cancers; (b) sensitivity, specificity, accuracy, and agreement between different assays identifying dMMR tumors; (c) lymphocytic infiltration in mCRPC samples; (d) mutational signatures and mutational load in metastatic prostate cancer; (e) the immune microenvironment in mCRPC; and (f) putative actionable immunotherapy targets in this disease. Our aim was to demonstrate that identification of dMMR with clinically available assays has limitations, and we hypothesized that a multipronged approach is necessary to adequately stratify mCRPC patients who could potentially benefit from immunotherapy with immune checkpoint–blocking drugs.

## Results

### dMMR mCRPC.

**Figure 1:**
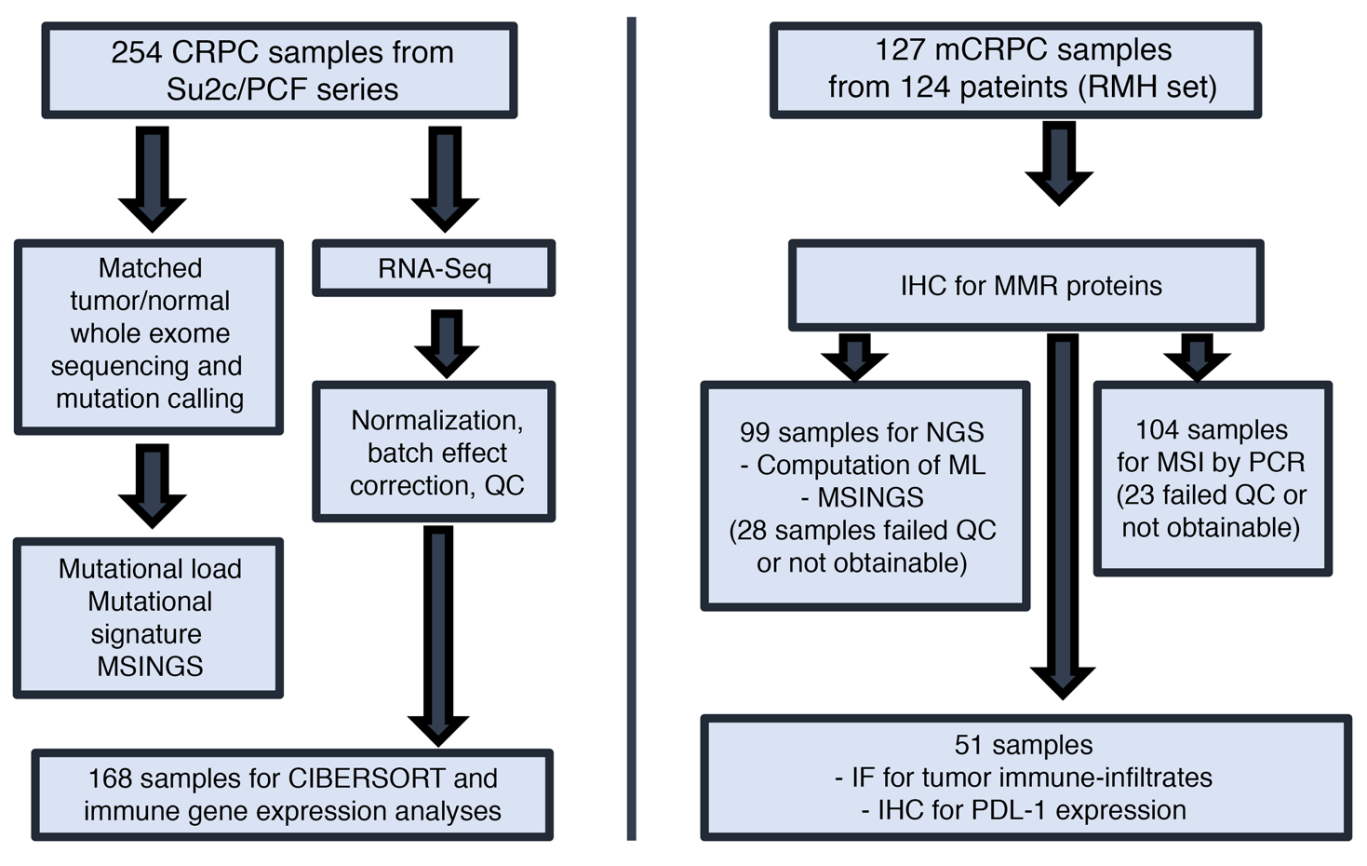
Consort diagram. Assays performed on 2 different cohorts of sample patients from the Royal Marsden Hospital (RMH) and the Stand Up To Cancer/Prostate Cancer Foundation (SU2C/PCF) database. ML, mutational load; QC, quality control.

Given the clinical need to identify and characterize dMMR tumors in APCs, we analyzed 127 mCRPC biopsies from a cohort of 124 mCRPC patients. For 85 patients, we had matched HNPC and CRPC samples (Figure 1). We first analyzed orthogonal assays for dMMR; these tests evaluated dMMR by (i) immunohistochemistry (dMMR_IHC); (ii) MSI by PCR (dMMR_MSI; Promega MSI Assay v1.2); (iii) targeted NGS of MMR pathway gene coding sequences (dMMR_MUT); (iv) MSI by NGS (dMMR_MSINGS). Overall, 10 patients had at least 1 tumor biopsy identified as having dMMR by IHC and/or MSI (8.1%, 10/124), and considered biomarker positive, with some patients having discordant results. Patient characteristics in the dMMR group and the comparator group were not dissimilar (Table 1).

**Figure 2:**
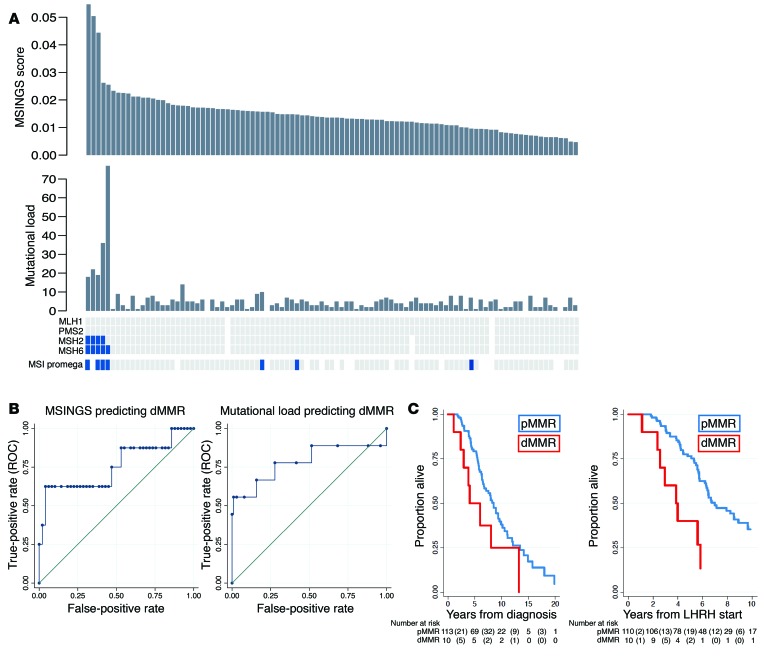
Comparative orthogonal analyses of dMMR in mCRPC. (**A**) Methods for detecting dMMR in 127 CRPC tumors from 124 patients with NGS available (samples sorted by MSINGS). From top to bottom: MSI by NGS (dMMR_MSINGS); mutational load per panel after SNP filtration; IHC for MLH1, PMS2, MSH2, MSH6 (blue marks absence of the protein); dMMR_MSI by PCR in blue. White indicates samples not assessable for analysis. Results for 1 dMMR patient are not shown, since MSINGS for this samples failed QC. (**B**) MSINGS score cutoff of 0.024 had sensitivity (SE) of 60% and specificity (SP) of 98% for predicting dMMR_IHC or dMMR_MSI, with an area under the ROC curve (AUC) of 0.79. ML ≥5.5 mutations had SE = 78% and SP = 72% for predicting dMMR_IHC or dMMR_MSI (AUC = 0.75). (**C**) Kaplan-Meier survival curves from diagnosis (left) according to MMR status (median OS [mOS], 8.5 years; interquartile range [IQR], 5.5–13.5 years for pMMR vs. 4.1 years; IQR, 2.9–8.0 years for dMMR; log-rank test *P* = 0.07). Kaplan-Meier survival curves from LHRH initiation (right), according to MMR status (mOS, 7.0 years; IQR, 5.3–13.5 years for pMMR vs. 3.8 years; IQR, 2.5–5.8 for dMMR; log-rank test, *P* = 0.003).

In view of concerns regarding discordance between dMMR_IHC and dMMR_MSI, we then evaluated 698 unstable (of 3,214) microsatellites present in our previously published targeted gene sequencing panel consisting of the coding regions of 113 genes (0.6-Mb panel) and estimated MSINGS (14, 15). These analyses revealed that prostate cancers with dMMR_IHC or dMMR_MSI often, but not always, have higher mutational loads and higher dMMR_MSINGS scores, with our data indicating overall that the dMMR_MSI Promega assay was most likely to give discordant, presumed false-positive, results. Comparisons between the different assays are presented in Figure 2A (cases ranked by MSINGS score). Critically, there was no easily defined cutoff for the MSINGS data dividing tumors that were definitely dMMR from other cancers. However, a cutoff of 0.0244 with this targeted MSINGS panel had an AUC of 0.79, a sensitivity of 60%, and a specificity of 98% to predict MMR cases defined positive by IHC and/or MSI (Figure 2B).

### Impact of mismatch DNA repair defects on outcome from prostate cancer.

The median overall survival (OS) for the dMMR_IHC/dMMR_MSI group was shorter than in the MMR-proficient (pMMR) group in univariate and multivariate analysis (3.8 vs. 7.0 years from start of luteinizing hormone–releasing hormone [LHRH]; adjusted hazard ratio (aHR), 4.09; 95% CI, 1.52–10.94; *P* = 0.005) as shown in Figure 2C and Table 2. Patients with pMMR and dMMR diseases were balanced in terms of clinical features, and no statistically significant differences were observed between the 2 groups in terms of radical treatments received, Gleason score, presence of metastatic disease at diagnosis, prostate-specific antigen (PSA), age, and stage at diagnosis (Table 1). Importantly, in this cohort of clinically aggressive tumors, 56% of the patients had metastatic disease at diagnosis.

### Intrapatient dMMR heterogeneity in primary disease.

Overall, for 85 patients we had both (matched, same patient) HNPC and CRPC samples available for analyses. Of these 85, 5 patients (5.88%) had evidence of IHC-negative foci within their primary disease samples acquired at diagnosis; 4 of these 5 (80%) had diffusely negative dMMR_IHC in mCRPC biopsies, with 1 patient progressing with MMR IHC–normal disease at mCRPC biopsy. Two of the 5 hormone treatment–naive prostate cancer (HSPC) samples with IHC-negative foci demonstrated the coexistence of IHC-positive prostate cancer, i.e., heterogeneous staining (Supplemental Figures 1 and 2; supplemental material available online with this article; https://doi.org/10.1172/JCI121924DS1). In contrast, a single CRPC sample had MMR protein IHC heterogeneity, these biopsies having been acquired from a large pelvic mass arising from a previously irradiated prostate. These data indicate that dMMR can be focal in primary disease, but that having dMMR in primary disease strongly associates with developing dMMR CRPC.

### PD-L1 expression and tumor-infiltrating lymphocytes in dMMR CRPC.

**Figure 3:**
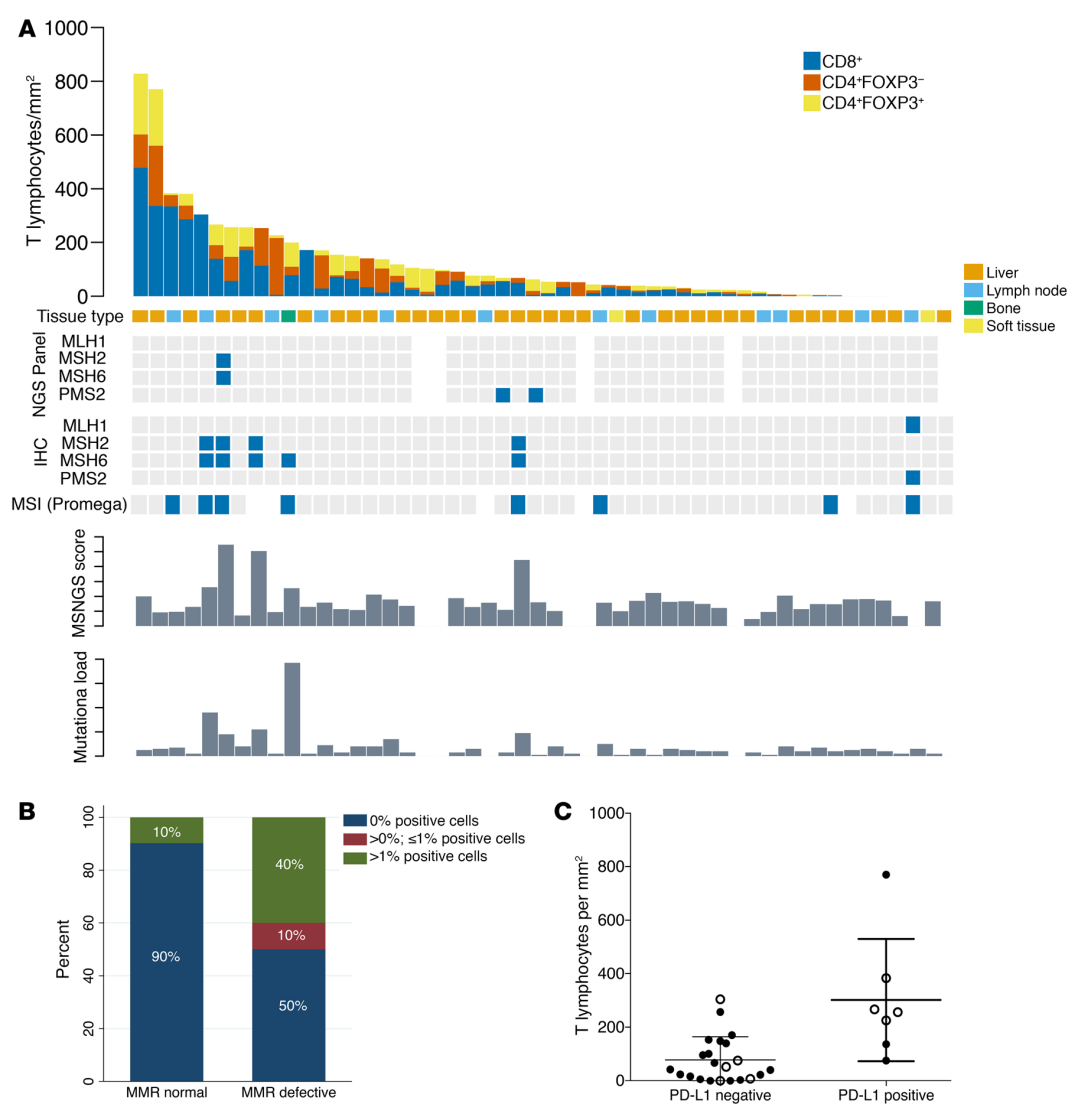
Tumor-infiltrating lymphocytes, molecular features, and PD-L1 expression of CRPC samples from RMH cohort. (**A**) Tumor-infiltrating T lymphocyte quantitation in 50 mCRPC biopsies, with MMR status according to the different orthogonal assays (MSI_MUT; MSI_IHC; MSI_MSINGS; mutation load), ordered from left to right by T cell infiltration score. A sample from 1 dMMR patient was not used for this analysis since it was a TURP sample taken at time of CRPC. Blue squares mark altered biomarker. (**B**) Stacked bar chart depicts proportion of PD-L1 immunohistochemical positivity (e.g., Supplemental Figure 3A) in samples reviewed by pathologists blinded to dMMR results in 51 mCRPC samples (*n* = 10 dMMR, *n* = 41 pMMR). (**C**) Dot plot showing the correlation between PD-L1 expression and T cell infiltration in mCRPC biopsies (*n* = 29). The *y* axis depicts total T cell infiltration defined as *n* of T cells/mm^2^ using a negative binomial regression model; there was an IRR of 3.91 (95% CI, 1.45–10.53; *P* = 0.007) for patients with PD-L1 > 0. Filled circles represent pMMR; open circles represent dMMR.

We next evaluated whether dMMR mCRPC is enriched for programmed death ligand 1 (PD-L1) (CD274) protein expression, given the key role of this protein in regulating anticancer immune responses. We performed PD-L1 IHC with a validated antibody to the PD-L1 carboxy terminal domain (Cell Signaling Technology) on 51 mCRPC biopsies, with a pathologist blinded to dMMR status scoring membranous staining in tumor cells (Supplemental Figure 3). Five of 10 (50%) dMMR mCRPC samples were scored as PD-L1 positive (Figure 3B), while 4 of 41 (9.8%) pMMR tumors had some positive PD-L1 staining. Although the optimal staining cutoff and optimal assay for determining PD-L1 expression as it pertains to therapeutic responses remain controversial, these data indicate a higher likelihood of PD-L1 positivity in dMMR mCRPC (mixed-effects logistic regression model odds ratio [OR], 14; 95% CI, 2–84; *P* = 0.005), providing further evidence for dMMR as a potential predictive biomarker for immune checkpoint inhibition in lethal prostate cancer.

We next quantified the density of tumor-infiltrating T lymphocytes (D-TILs) in biopsies from patients in this cohort for whom we had sufficient tumor tissue. D-TILs was here defined as numbers of CD4^+^ cells, with and without FOXP3, and CD8^+^ lymphocytes per mm^2^ of tumor determined through 180 multispectral, multicolor immunofluorescence (IF) image cubes (×200 magnification; median of 3 images per case; *n* = 51 selected CRPC biopsies). Tissue sites included lymph node biopsies (*n* = 35), bone (*n* = 12), liver (*n* = 2), soft tissue metastases (*n* = 1), and 1 sample from transurethral resection of the prostate (TURP; the last sample was excluded from this analysis, as it was not metastatic). T cell infiltration was strikingly heterogeneous, ranging from 0 to 828 lymphocytes/mm^2^ (e.g., Supplemental Figure 3, B and C). Ranking tumors by T cell density showed that 5 of the 9 (55.5%) dMMR_IHC/dMMR_MSI cases were allotted to the upper quartile of D-TILs in this cohort and 3 of these 5 cases had more than 10 mutations (>90th percentile; 113-gene panel). The remaining 4 dMMR_IHC/dMMR_MSI cases, however, did not show increased D-TILs relative to this cohort. These data suggest that some, but not all, mCRPC with dMMR_IHC/dMMR_MSI have higher D-TIL levels than tumors without dMMR (Figure 3A). Of the remaining pMMR samples in the upper quartile of D-TILs, none had pathogenic DNA repair defects by our targeted NGS panel; 2 pMMR samples in this group with high D-TIL levels showed deleterious mutations in other pathways (PIK3CA E542K; JAK1 E1051*). Overall, in this cohort of tumors analyzed for D-TILs, PD-L1 expression was associated with increased T cell infiltration in mCRPC samples (incidence rate ratio [IRR], 3.91; 95% CI, 1.45–10.53; *P* = 0.007; Figure 3C).

### DNA mutation signatures and mutation load (SU2C/PCF cohort): DNA repair defects.

**Figure 4:**
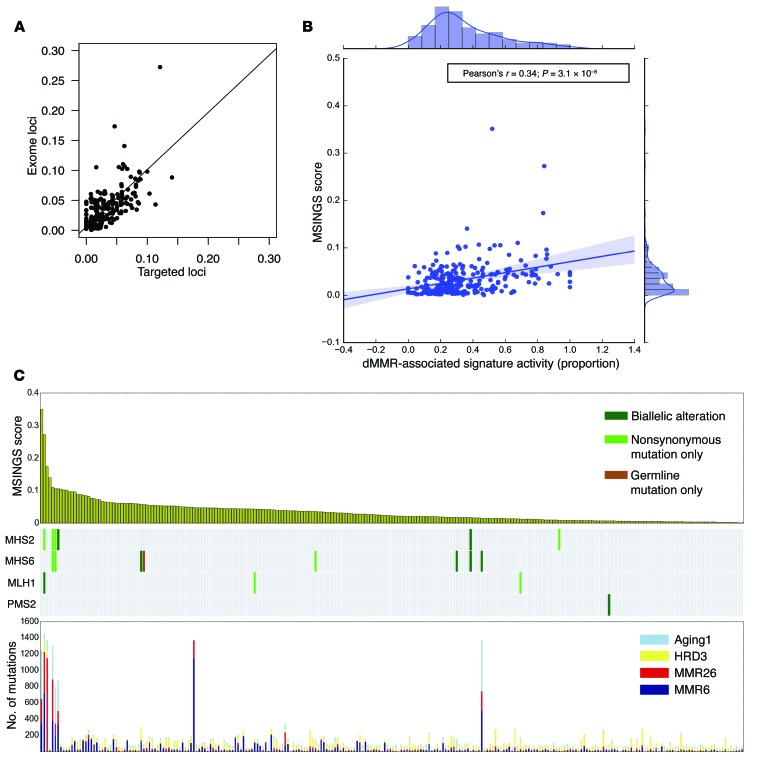
Immune and mutational signature characterization of mCRPC in the SU2C/PCF dataset (*n* = 254). (**A**) Correlation between MSINGS by targeted panel and by exome sequencing. (**B**) Association between MSINGS score and dMMR signature activity. (**C**) MSINGS score (top), MMR gene mutations (middle), and DNA mutational signature activity (bottom). MMR-dominant indicates tumors with >50% dMMR-related mutations. Biallelic loss-of-function (LOF) events (homozygous deletions, nonsynonymous mutations + LOH, or multiple nonsynonymous mutations) (*n* = 7), single-allele nonsynonymous mutations (*n* = 6), or germline mutations (*n* = 1) in canonical MMR genes (MSH2/6, MLH1, PMS2) are indicated (for details, see Supplemental Table 1).

We next evaluated the genomic and immunological features of mCRPC by analyzing exomes acquired from mCRPC biopsies by the SU2C/PCF International Prostate Cancer Dream Team (Figure 1). We first demonstrated that MSINGS generated by targeted panel analyses correlated with MSINGS acquired by analyzing exome sequencing data (*r* = 0.73, *P* < 0.0001) (Figure 4A). Surprisingly, in 254 exomes, as with the targeted NGS efforts, there was no clear MSINGS cutoff for MMR tumors, although tumors with detected MMR gene mutations frequently had the highest MSINGS score (Figure 4B).

Beyond mutations in canonical MMR genes, we hypothesized that signatures of mutational processes in individual tumors would shed light on DNA repair deficiencies (10, 16, 17) that may associate with distinct immunologic subtypes. To assess this, two independent groups from our team applied two different mathematical models to identify the mutational signatures present in each mCRPC biopsy utilizing either (i) Bayesian non-negative matrix factorization (NMF) (18) or (ii) non-Bayesian NMF (19). We identified 4 dominant mutational signatures that matched known Catalogue of Somatic Mutations in Cancer (COSMIC) mutational signatures, including dMMR-associated signatures (MMR6 and MMR26 matching to COSMIC signatures 6 and 26), homologous recombination deficiency–associated (HRD-associated) signatures (HRD3 matching to COSMIC signature 3), and aging-associated signatures (Aging1 matching to COSMIC signature 1), with cosine similarities of 0.96, 0.88, 0.89, and 0.99, respectively. Patients with germline mutations (*n* = 1), nonsynonymous somatic mutations (*n* = 6), or biallelic events (*n* = 7) in canonical MMR genes (total *n* = 14) had higher dMMR-associated DNA mutational signature activity (Supplemental Figure 4, A–C), which correlated with higher dMMR-associated mRNA expression signatures (Supplemental Figure 5, A and B). Patients with high MSINGS had predominant dMMR DNA mutation signatures (Figure 4C), and there was a strong correlation between dMMR mutational signature activity and MSINGS score (Figure 4B). Interestingly, however, some tumors without variants in MMR genes and with low MSINGS scores also had some evidence of dMMR-associated DNA mutation signatures. The significance of these dMMR signatures in such tumors is unclear.

### Immune transcripts in metastatic prostate cancer (SU2C/PCF cohort).

**Figure 5:**
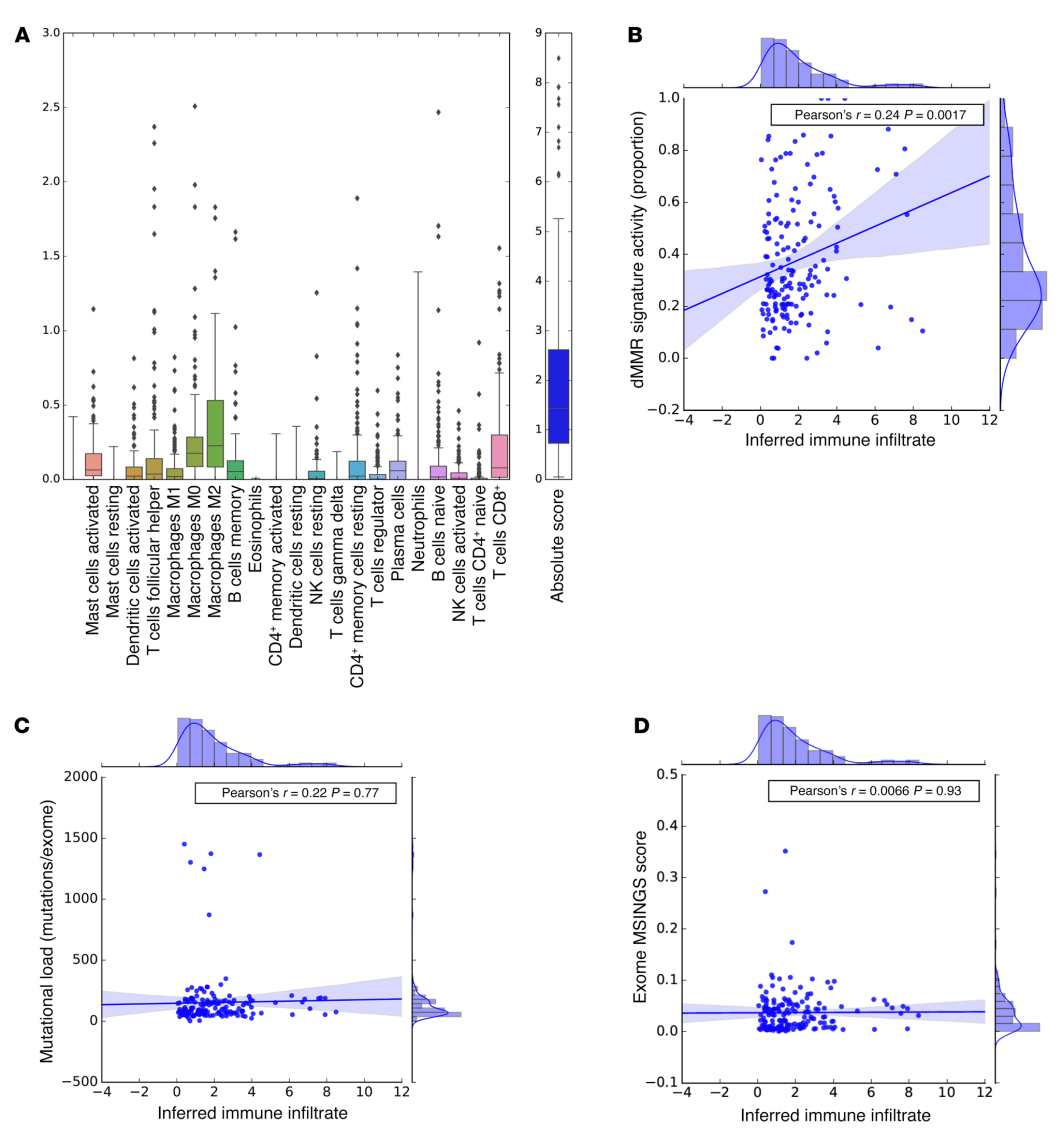
CIBERSORT analyses quantifying 22 immune cell subtypes and overall inferred immune infiltrate in mCRPC tumors with available transcriptomes from the SU2C/PCF dataset (*n* = 168). (**A**) The *y* axis is an absolute quantification. We observed overall increased levels of M2-like macrophage signature relative to M1-like macrophages. (**B**) Association of dMMR mutational signature activity (proportion) with inferred immune infiltrate; the inferred Pearson’s correlation coefficient is 0.24 (*P* = 0.0017). (**C**) Association of mutational load with inferred immune infiltrate (Pearson’s ρ = 0.02, *P* = 0.77). (**D**) Association of MSINGS scores with immune infiltrate (Pearson’s ρ = 0.0066, *P* = 0.93).

**Figure 6:**
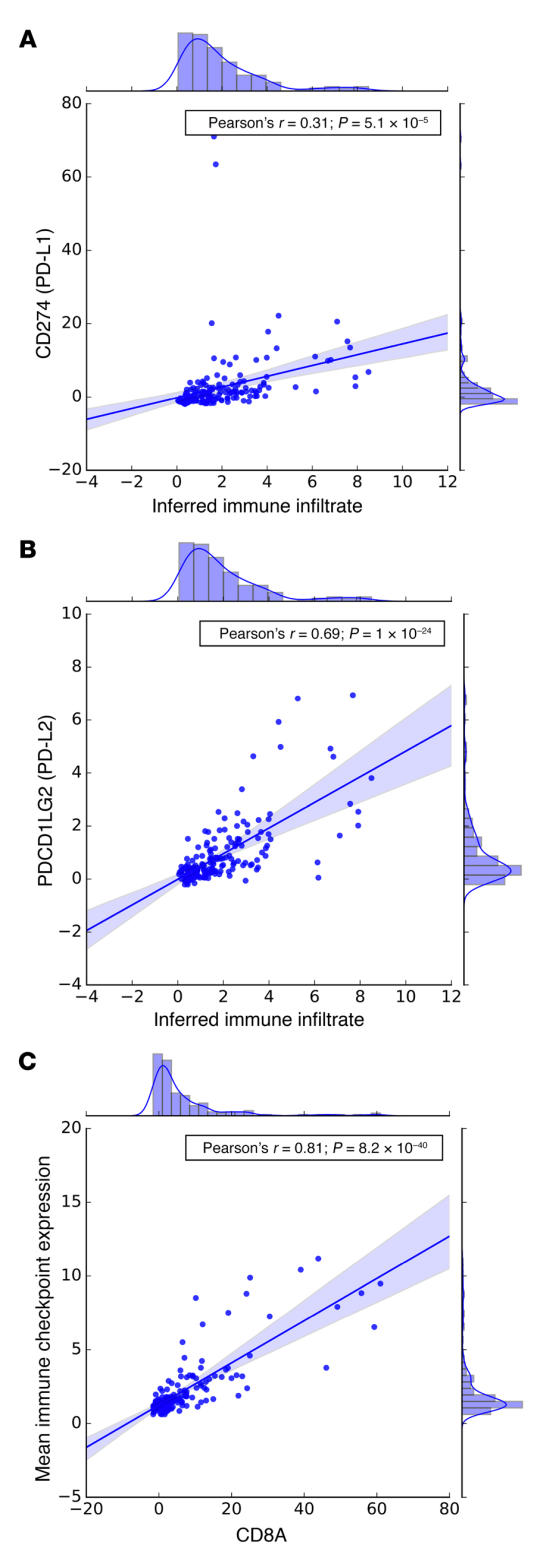
Analyses of immune cell and immune checkpoint transcripts from the SU2C/PCF dataset (*n* = 168). Correlation between inferred immune infiltrate and (**A**) PD-L1 and (**B**) PD-L2 expression in mCRPC transcriptomes. (**C**) Strong correlation between CD8A expression and the geometric mean of the other 31 immune checkpoint-related genes (Pearson’s ρ = 0.81, *P* = 8.2 × 10^–40^).

To interrogate the relationship between cancer genomics and the immune landscape, we next analyzed matched transcriptomes from 168 tumors (Figure 1) using CIBERSORT (20), a method developed to deconvolute immune cell populations from bulk transcriptome data using immune cell–associated signatures. From these data, we inferred overall immune infiltrate and relative immune cell populations in mCRPC biopsies (Figure 5A), and observed substantial variation in overall immune infiltrate–related transcripts among tumor biopsy sites, as well as heterogeneity in inferred immune cell populations. Overall, monocytes and macrophages were the most common inferred immune cell populations, with higher levels of M2-polarized versus M1-polarized macrophages. These relationships persisted when we examined bone and lymph node metastases separately (data not shown). Notably, the proportion of dMMR mutational signature activity, but not overall mutation load or MSINGS score, was positively associated with inferred total immune infiltrate based on transcriptome data (Pearson’s *r* = 0.24; *P* = 0.002; Figure 5, B–D). This association persisted when stratifying by biopsy site (bone metastases, *r* = 0.36, *P* = 0.008; lymph node metastases, *r* = 0.24, *P* = 0.05). Interestingly, inferred immune infiltrate also correlated with PD-L1 (Pearson’s *r* = 0.31; *P* = 5.1 × 10^–5^) and PD-L2 (Pearson’s *r* = 0.69; *P* = 1 × 10^–24^) mRNA expression (Figure 6, A and B).

### Immune transcript landscape in dMMR CRPC transcriptomes (SU2C/PCF cohort).

**Figure 7:**
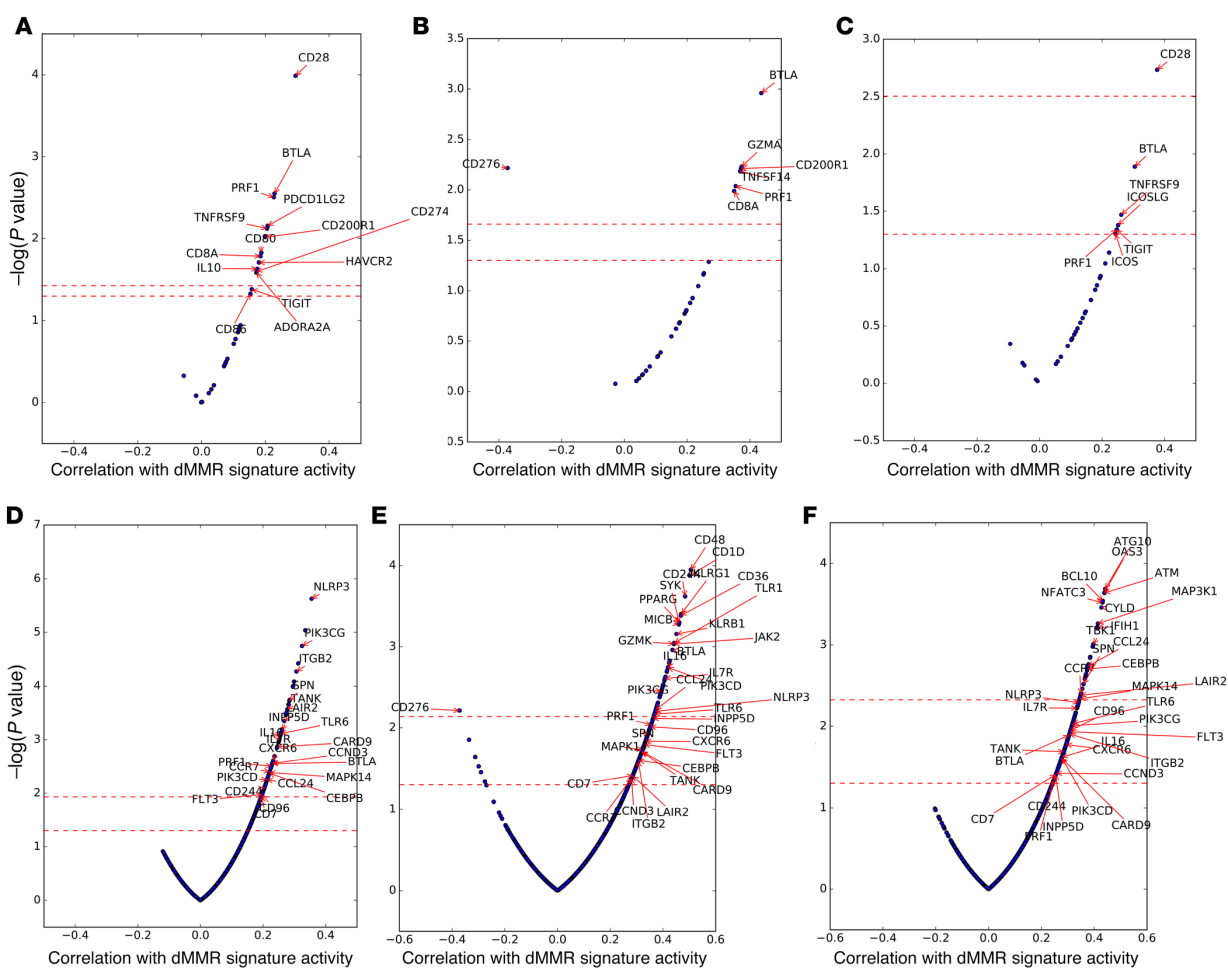
Immune transcripts associated with dMMR mutation signature activity in mCRPC tumors from the SU2C/PCF dataset (*n* = 168). (**A**) Expression of immune checkpoint–related genes associated with dMMR mutation signature activity (32 immune checkpoint genes analyzed; Overall). (**B**) Expression of immune checkpoint–related genes associated with dMMR mutation signature cancers (32 immune checkpoint genes analyzed; Bone Metastases). (**C**) Expression of immune checkpoint–related genes associated with dMMR mutation signature activity (32 immune checkpoint genes analyzed; Lymph Node Metastases). (**D**) Discovery of immune mRNA transcripts associated with, in RNA-Seq analyses, dMMR mutation signature activity (762 immune transcript NanoString panel; Overall). (**E**) Discovery of immune mRNA transcripts associated with, in RNA-Seq analyses, dMMR mutation signature activity (762 immune transcript NanoString panel; Bone Metastases). (**F**) Discovery of immune mRNA transcripts associated with, in RNA-Seq analyses, dMMR mutation signature activity (762 immune transcript NanoString panel; Lymph Node Metastases).

We next examined the expression levels of a curated set of genes associated with immune checkpoints (21) (*n* = 32; see Methods). The overall geometric mean of expression of these transcripts strongly correlated with CD8A expression (Figure 6C) in these mCRPC biopsies. We next correlated the expression of these 32 immune checkpoint–related genes with dMMR-associated mutational signature activity. To examine consistent associations of dMMR signatures with immune checkpoint gene expression independent of biopsy site (distribution in Supplemental Table 2), we focused our primary analysis on genes that passed multiple hypothesis testing (FDR < 0.1) in the overall analysis as well as statistical significance testing (*P* < 0.05) when stratified by biopsy site. This approach filtered out genes associated only in specific metastatic settings. Only 2 genes met these stringent criteria: the immune checkpoint molecule *BTLA* and the cytolytic molecule *PRF1* (Figure 7, A–C). In bone metastases, the inhibitory myeloid receptor *CD200R1* and the CD8^+^ T cell molecules *CD8A* and *GZMA* were also associated with dMMR signature activity, while CD276 (also known as B7-H3) was strongly negatively associated (FDR, <0.1) (Figure 7B). Interestingly, CD276 expression was the only immune gene strongly positively associated with the HRD mutational signature (FDR, <0.1). This association of B7-H3 expression with HRD was confirmed at a protein level by IHC (data not shown). When significance in each biopsy site was not required, the expression of 12 immune checkpoint genes was correlated with dMMR mutational signature activity (FDR, <0.1): *CD28*, *CD200R1*, *BTLA*, *PRF1*, *TNFRSF9* (*4-1BB*), *ADORA2A* (*A2A receptor*), *PDCD1LG2* (*PD-L2*), *CD8A*, *IL10*, *CD80* (*B7-H1*), *HAVCR2* (*TIM-3*), and *CD274* (*PD-L1*).

We then applied this approach to an exploratory analysis of 762 immune-related genes (22) (Figure 7, D–F) in order to discover transcripts differentially overexpressed or underexpressed in dMMR tumors. We hypothesized that such transcripts might have a key role to play in anticancer inflammation and, if rigorously validated, could yield not only a better understanding of these processes, but novel anticancer therapeutic strategies. Overall, 89 genes were associated with dMMR signature activity after adjustment for multiple hypothesis testing when considering all biopsy sites (Figure 7D and Supplemental Table 3); 55 and 36 genes were associated in bone metastases and lymph node metastases, respectively (Figure 7, E and F, and Supplemental Tables 4–6). With the rigorous filters described above applied, 24 genes were consistently correlated with dMMR signature activity (Figure 7, D–F). This broader analysis suggested that dMMR may be associated with a more complex immune infiltrate; upregulated transcripts included genes generally associated with dendritic cells (FLT3), macrophages/myeloid cells (PIK3CG), and T cells (CD8A, BTLA).

## Discussion

Linking genomics to the immunologic features of tumors is of major interest in the field of immunooncology. In May 2017, the FDA granted accelerated approval for the anti-PD1 monoclonal antibody pembrolizumab — the first tissue-agnostic antitumor agent to be approved for solid malignancies underpinned by dMMR (3, 23). However, emerging clinical data show that (a) dMMR cancers do not always respond to immunotherapy; and (b) cancers responding to immune checkpoint targeting are not necessarily dMMR as defined per conventional IHC and PCR assays (3, 23, 24). To improve the understanding of dMMR in prostate cancer, our work provides the first integrated analyses to our knowledge of genomic, transcriptomic, and clinical data of large cohorts of advanced, castration-resistant prostate cancer. Among the clinically important findings, we demonstrate (a) that dMMR prostate cancer represents a clinically aggressive phenotype; and (b) substantial discordance between orthogonal approaches to detecting dMMR, including clinical assays that are standard in other disease settings. To overcome the limitations presented by these assays, we leveraged NGS data from WES and mapped the genomic consequences of dMMR. We show that 2 distinct dMMR-associated mutational signatures can be robustly derived from exomes and that these signatures predominate in cancers with MMR gene mutations, and associate with higher MSINGS and previously described transcriptomic signatures of MMR-defective disease. Although mutational signatures are powerful alternate readouts for dMMR, these data need to be interpreted cautiously, since limitations to use include the similarities/overlapping of dMMR signatures with other signatures, limited precision in low-mutational load tumors, and limited feasibility for most targeted sequencing approaches.

Using NGS data, hundreds of microsatellites can be queried at a time, potentially improving the chances of MSI detection. Using MSINGS analysis of all satellites in exomes, we show that a high MSINGS score associates with a high proportion of dMMR signatures. Next, by delineating satellites for MSINGS analysis in our targeted sequencing panel, we show a strong correlation between MSINGS score from exomes and MSINGS score from targeted sequencing, indicating that MSINGS scores can be reliably obtained from these and can provide a robust tool for identifying prostate cancer underpinned by dMMR. A major challenge, however, remains determining the cutoff point in the MSINGS score that determines the presence of a dMMR tumor. Our data indicate that a cutoff that clearly discriminates dMMR cancers may be difficult to define.

Finally, using transcriptomic analysis, we conducted a comprehensive study of the immunological consequences of dMMR in mCRPC. Our data showed that prostate cancers with prominent dMMR mutational signatures have higher inferred immune cell infiltration. Qualitatively, dMMR mutational signatures associated with increased T cell–related transcripts, as well as immune checkpoint–related transcripts including PD-L1 and PD-L2. Interestingly, dMMR tumors had higher expression of factors involved in T (and NK) cell recruitment (CCRs and CXCRs) and function (PRF1, BTLA, and TNFRSF9/CD137), with multiple genes related to immune checkpoints, including CD200R1 and the metabolic immune checkpoint adenosine receptor 2A (ADORA2A), also being upregulated (Supplemental Table 6). Our detailed analysis of immune-related genes in dMMR tumors also revealed prominent expression of markers attributable to myelomonocytic cells (Supplemental Table 6), including VCAM1, NLRP3, and JAK2, described to mediate the accumulation and expansion of myeloid-derived suppressor cells (MDSCs). Our results also identified as relevant the expression of CD36, a protein reported to be critical for M2 macrophage activation, and PI3Kγ, which was recently reported to be involved in the immunomodulatory activity of tumor-associated myeloid subsets. Indeed, inhibition of PI3Kγ in myeloid cells has been reported to reverse resistance to checkpoint blockade therapy in preclinical models. These mRNA data suggest that in some dMMR mCRPCs, the efficacy of immune checkpoint blockers may be enhanced through combination strategies aimed at depleting myeloid tumor subsets. The metabolic target ADORA2A is also of interest, as it is relatively overexpressed on both suppressive myeloid cells and Tregs. Determining the relative roles of these potential targets in dMMR mCRPC now requires rigorous translational evaluation in biology-driven therapeutic trials.

In conclusion, our data show that a subset of lethal prostate cancers is underpinned by dMMR defects. We show that dMMR is usually present at diagnosis, and our data indicate that these tumors constitute a discrete subtype with decreased survival time, with only a proportion of cases having high mutation load and PD-L1 IHC staining. dMMR exome mutational signatures and high MSINGS scores are associated with complex immune mRNA profiles that may require further sub-classification based, for example, on the degree of myeloid cell infiltration, which can affect clinical behavior and responses to immune checkpoint therapies.

## Methods

### Patients

We analyzed data from 2 cohorts of men with CRPC: (i) an updated combined cohort of men with mCRPC from multiple institutions comprising the SU2C/PCF Prostate Cancer Dream Team and (ii) a cohort of men with CRPC referred to the Royal Marsden, whose diagnostic samples and/or mCRPC biopsies were molecularly characterized between January 2015 and June 2016 at the Institute of Cancer Research (London, United Kingdom). Patients were included in this study if they had available formalin-fixed, paraffin-embedded (FFPE) tissue samples from metastatic sites or primary tumors for MMR panel testing by IHC. Diagnostic tissue was obtained from prostate needle biopsy, TURP, or prostatectomy procedures. CRPC tissue was obtained from metastases within bone, lymph node, soft tissues, or visceral organs. All tissue blocks were sectioned and only considered for IHC analyses if adequate material was present (≥50 tumor cells; reviewed by a pathologist in our group). Patients with histologic features supporting a diagnosis of pure neuroendocrine or small cell cancer were not included. Demographics and clinical data were retrospectively collected from hospital electronic patient records.

### IHC

FFPE samples were cut in 3-μm sections onto charged glass slides. Immunostaining was performed with antibodies against MSH2, MSH6, MLH1, PMS2 (M3639, clone FE11, 1:50; M3646, clone EP49, 1:500; M3640, clone ES05, 1:100; and M3647, clone EP51, 1:100; Dako, Agilent Technologies), and PD-L1 (catalog 13684, clone E1L3N, 1:200; Cell Signaling Technology). Heat-induced antigen retrieval was performed using Tris-EDTA buffer, pH 8.1, in a MenaPath Antigen Access Unit at 125°C for 2 minutes to detect MSH2, MSH6, MLH1, and PMS2 or microwave oven for 18 minutes for the detection of PD-L1. Endogenous peroxidase was inactivated using 3% H_2_O_2_, and nonspecific staining was blocked using protein block serum-free solution (X0909, Dako, Agilent Technologies). Detection by diaminobenzidine reaction was performed using a Dako REAL EnVision Detection System (K5007, Agilent Technologies).

Scoring of MSH2, MSH6, MLH1, and PMS2 was achieved by segregating cases in a binary fashion between positives and negatives using College of American Pathologists criteria for biomarker reporting in colorectal carcinomas (25). In brief, nuclear staining–positive tumors, regardless of intensity, were called positive, and cases with absent nuclear staining were called negative. A comment on heterogeneity of staining was made when juxtaposition between positive and negative areas was observed with adequate internal controls. PD-L1 staining was done using a PD-L1 monoclonal antibody (rabbit, clone E1L3N, 1:200; Cell Signaling Technology), which has recently been shown to be comparable with other, FDA-approved IHC assays (26). Heat-based antigen unmasking was achieved by microwaving the slides in Tris-EDTA buffer, pH 8.1. The antibody was diluted at 1:200 and incubated at room temperature for 1 hour. Reactions were visualized using the Dako REAL EnVision Detection System (Agilent Technologies). Partial or complete membrane staining was considered a signal and cases were evaluated as a tumor proportion score, i.e., number of signal positive viable tumor cells/total number of viable tumor cells as previously described (27).

### IF

Multiplex sequential IF staining was performed on 3-μm sections from FFPE tissue. Antigen retrieval was performed using CC1 buffer (950-224, Ventana Medical Systems) at 98°C for 36 minutes in a water bath. Endogenous peroxidase was inactivated in 3% H_2_O_2_ for 10 minutes. Tissue sections were incubated for 60 minutes at room temperature with antibodies against CD4 (104R-16, clone SP35, 1:100, Cell Marque) and CD8 (M7103, clone C8/144B, 1:200, Dako, Agilent Technologies). A second layer of antibodies using Alexa Fluor 555–conjugated IgG (H+L) goat anti-rabbit (A21429, Invitrogen) and Alexa Fluor 488–conjugated IgG (H+L) goat anti-mouse (A-11029, Invitrogen) was used to detect CD4 and CD8, respectively. Tissue sections were treated with an avidin/biotin blocking kit according to the manufacturer’s protocol (ab64212, Abcam) and rabbit/mouse normal serum at 5% for 30 minutes. Next, tissue sections were incubated for 60 minutes with antibodies against FOXP3 conjugated to biotin (13-4777-82, clone 236A/E7, 1:100, eBioscience) and EpCAM conjugated to Alexa Fluor 647 (5447S, clone VU1D9, 1:200, Cell Signaling Technology). Tissue sections were incubated with streptavidin peroxidase (HRP) (K5001, Dako, Agilent Technologies) for 15 minutes, followed by a TSA Coumarin detection system (NEL703001KT, PerkinElmer) for 10 minutes. Nuclei were counterstained with DRAQ7 (DR71000, BioStatus), and tissue sections were mounted with ProLong Gold antifade reagent (P36930, Molecular Probes). After staining, slides were scanned using a multispectral camera provided by the Vectra (PerkinElmer) system (28). Whenever possible, more than one nonoverlapping micrograph at ×20 magnification was collected.

### Digital image analysis

Linear unmixing of multispectral images was done using inForm Cell Analysis software version 2.1.1. A tissue segmentation algorithm was developed using EpCAM positivity as a tumor mask to separate neoplastic cells from adjacent stroma. A cell segmentation algorithm was developed using DRAQ7 as nuclear marker and phenotype determination was based on staining for EpCAM, CD4, FOXP3, and CD8. Cells in tumor areas selected by the algorithm were separated into bins as follows: CD4^+^FOXP3^–^ cells, CD4^+^FOXP3^+^ cells, CD8^+^ cells, and EpCAM^+^ tumor cells. All tissue segmentation, cell segmentation, and phenotype determination maps were reviewed by a pathologist in our group. For each image, the tumor area (in mm^2^) and the number of CD4^+^FOXP3^–^, CD4^+^FOXP3^+^, and CD8^+^ cells were determined to calculate the lymphocytic D-TILs (LD-TIL) determined as: (∑ T lymphocytes from all images)/(∑ of areas from all images).

### MSI status

DNA (1 ng) was amplified using the MSI Analysis System (Promega) according to the manufacturer’s protocol. The MSI Analysis System is composed of 7 fluorescently labeled microsatellites including 5 mononucleotide repeat markers (BAT-25, BAT-26, NR-21, NR-24, and MONO-27) for detecting MSI and 2 pentanucleotide repeat markers (Penta C and Penta D) for sample identification. The PCR products were run in an ABI 3730 DNA Analyzer and subsequently analyzed using GeneMapper 4.0 software (Thermo Fisher Scientific). Samples with microsatellite instability in 2 or more loci were defined as MSI-high, whereas samples with a single locus were defined as MSI-low; samples were microsatellite-stable (MSS) if no instability at any of the loci tested was detected. For the purpose of statistical analysis, cases were dichotomized between MSI-high and MSS/MSI-low.

### Sequencing and bioinformatics

#### Mutation load and calls.

WES analysis was performed using standard analytical pipelines (29), including human genome alignment (30), somatic mutation analysis (31), and quality control (12). For panel testing data, the mutation load was extracted from targeted NGS panel data after spurious and germline changes were filtered out using previously described methods (32). Genetic variants were called using the GATK pipeline (30). Low-quality variants were removed (haplotype score >200, mapping quality <40, coverage depth <60, alternative allele <5% of reads, multiallelic calls, indels, known poorly sequenced sites). Variants were then annotated using Oncotator (version 1.8.0) (33). Germline variants were defined when the allele frequency was more than 5% in our cohort (*n* = 127) or in 2 or more public databases (ExAC, ref. 34; 1000 Genomes, ref. 35; and dbSNP, ref. 36) or with more than 99.9% of the reads being the alternate allele. These germline variants were filtered out. Finally, point mutations described as somatic in the COSMIC database (37) at least 10 times were then “added” back into the mutation count. For small indels the same filtering was used, and copy number information was obtained using CNVkit v0.3.5 (38); these were combined with filtered SNPs to find samples with somatic gene loss for MMR, DSB, NER, and BER genes.

#### MSINGS.

MSINGS software (13) was used to score samples for an MSI-like phenotype by assessing targeted next-generation DNA-sequencing data; due to batch-related exome sequencing variability, we were unable to utilize MSINGS on these data. In brief, the algorithm functions by (a) identifying possible DNA repeat regions; (b) examining the frequencies of these alleles bearing varying repeat lengths; and (c) comparing these values with a baseline reference from MMR-intact specimens. Our targeted panel contained 3,214 possible loci. Tumor and normal samples were used produce an enriched reference set of 698 loci that could be utilized to predict MSI status computationally. We also used the default setting to derive MSINGS from WES data (tumor-normal paired), which were scaled by a factor of 0.209 for comparison with targeted panel scores.

#### Targeted NGS.

Targeted sequencing was performed as previously described (15). Libraries were constructed from 40 ng DNA using GeneRead Mix-n-Match V2 (QIAGEN) customized 113 genes panel, and pooled libraries were sequenced on the MiSeq (Illumina). FASTQ files were generated using Illumina MiSeq Reporter v2.5.1.3. Sequence alignments were performed using Burrows-Wheeler Aligner (BWA) tools and the Genome Analysis Toolkit (GATK) variant annotator by the QIAGEN GeneRead Targeted Exon Enrichment Panel Data Analysis portal.

#### Exome mutational signatures.

Mutational signatures were derived by assessing types of somatic mutation and the nucleotides immediate upstream and downstream of the mutations (96 base substitutions in trinucleotide sequence contexts) using 2 methods analyzed independently: (i) Mutation Signature Profiling (a Bayesian non-negative matrix factorization [NMF] method) using default parameters (18) (http://software.broadinstitute.org/cancer/cga/msp); and (ii) a non-Bayesian approach using multiplicative updates (19) with the Brunet method (39). The optimal rank (number of mutational signatures) was inferred after manually examining cophenetic coefficients, residuals, and residual sum of squares for 50 NMF runs at ranks 2–8, as well as comparing discovered signatures with previously discovered signatures using a cosine similarity measure. High cophenetic coefficients, low residuals, low residual sum of squares, and high cosine similarity to previous signatures were preferred. We used the R-packages SomaticSignatures (40) V2.6.1 and NMF (41). Since NMF is nondeterministic, we performed 200 independent NMF runs for a given rank and chose the resulting mutational signatures and signature activity per tumor from the NMF run with the minimum residual error. Linear regression was used to assess the mutational signature–associated gene expression. The correlation coefficient was used for Gene Set Enrichment Analysis (GSEA) (pre-ranked gene list; http://software.broadinstitute.org/gsea/) with the default parameters.

### RNA-Seq analysis

Available RNA-Seq data from the SU2C combined cohort (12) were analyzed. Expression data were examined and adjusted for batch effects using ComBat (42) via the R Bioconductor package “sva” V3.22.0 (43). Data in BAM format have been deposited into the NCBI’s dbGaP database (phs000915.v1.p1; see methods in ref. 12 and ref. 44 for details).

Correlations and associated *P* values between immunomodulatory gene expression, mutational load, and mutational signature activity were calculated using Pearson’s correlation. A Benjamini-Hochberg FDR of 0.1 was used to identify significantly correlated genes. For significant correlations, individual scatterplots were manually examined, outliers were removed, and the significance of the correlation was verified.

Tumor sample immune infiltrate was quantified using CIBERSORT (20), which was run using the CIBERSORT interface (https://cibersort.stanford.edu) set to absolute quantification output. Gene-level transcripts per million (TPM) was used as input, and LM22 (20) (leukocyte gene signature matrix) was used to deconvolve 22 immune cell subset populations. Correlations between immune cell subsets, mutational load, and mutational signatures were calculated as above.

Immune checkpoint genes along with other markers of T cell infiltrate and activity (*n* = 32 total) whose gene expressions we examined were *CD28*, *ICOSLG*, *ICOS*, *TNFRSF9*, *TNFSF9*, *TNFSF4*, *TNFRSF4*, *CD70*, *CD27*, *CTLA4*, *PDCD1*, *CD274*, *PDCD1LG2*, *CD47*, *HAVCR2*, *LGALS9*, *ADORA2A*, *CD200*, *CD200R1*, *CD276*, *VTCN1*, *TNFSF14*, *BTLA*, *TIGIT*, *CD8A*, *PRF1*, *IL10*, *CD80*, *GZMA*, *CD86*, *IFNG*, and *LAG3* (21). We calculated a geometric mean of expression levels across the 32 genes to generate an overall measure of immunomodulatory gene expression. For comparison of overall immunomodularity gene expression with CD8A (Figure 6C), the geometric mean of the other 31 genes was calculated.

### Statistics

OS was measured from the date of diagnosis, and date of start of LHRH agonist alone or with anti-androgen for metastatic or advanced disease, to the date of date of death from any cause. The relationship between dMMR_IHC and MMR_MSI tumors and OS was analyzed using univariate and multivariate Cox’s regression modeling, adjusting for radical treatment (prostatectomy or radiotherapy), Gleason score, age, PSA and nodal status, stage, and presence of metastatic disease at diagnosis. One or more factors were missing in approximately 16% of patients, and these were considered to be missing at random. Multiple imputation by chained equations with the above coefficients was used to generate 20 imputations; per-imputation estimates were combined using Rubin’s rules. Youden’s statistic (45) was used to determine the optimal cutoff for MSI by NGS and mutational load. To determine the statistical association between PD-L1 staining and dMMR, we applied a mixed effects logistic regression model with a random intercept effect per patient, which accounts for the correlation between samples from the same patient. All statistical tests were 2-sided. A *P* value less than 0.05 was considered significant.

### Study approval

All SU2C/PCF study individuals provided written informed consent for collection of fresh tumor biopsies and for comprehensive molecular profiling of tumor and germline samples. All Royal Marsden Hospital patients gave written informed consent and were enrolled in institutional protocols approved by the Royal Marsden NHS Foundation Trust Hospital (London, UK) ethics review committee (reference no. 04/Q0801/60).

## Author contributions

DNR, PR, DL, JSdB, and EMVA designed the research; DL, WY, SC, JM, GS, and M. Clarke coordinated overall sequencing and bioinformatics analysis; RR, S. Miranda, AF, IF, and LSTM were involved in pathology review; M. Crespo carried out IF analysis; MBL carried out microsatellite instability analysis; DD, CB, GB, AS, DK, SA, KPB, KM, S. Salipante, CL, and ADA analyzed data and provided analytical tools; S. Sumanasuriya, MSF, DB, ZZ, CM, DM, and S. Mullane acquired data and were clinical contributors; DNR, PR, and DL wrote the manuscript, which all the authors reviewed; JSdB and EMVA provided funding; WY, AA, CGD, JSdB, and EMVA supervised the work; MTS, CCP, MET, HB, MAR, M. Cieslik, DR, EH, NS, JA, WA, HS, and PSN are SU2C-PCF Dream Team principals, and CLS and AMC are Dream Team co-leaders.

## Supplementary Material

Supplemental data

ICMJE disclosure forms

Supplemental Tables 1-6

## Figures and Tables

**Table 2:**
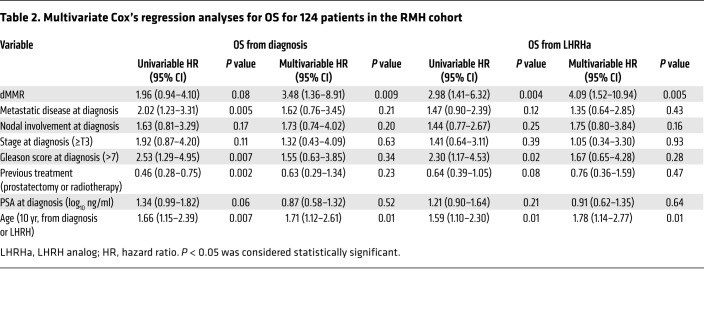
Multivariate Cox’s regression analyses for OS for 124 patients in the RMH cohort

**Table 1:**
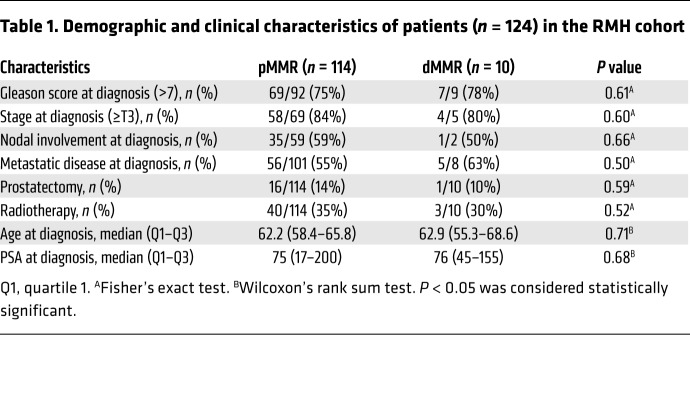
Demographic and clinical characteristics of patients (*n* = 124) in the RMH cohort

## Version 1 09/04/2018

Electronic publication

## Version 2 10/01/2018

Print issue publication

## References

[1] Kwon ED (2014). Ipilimumab versus placebo after radiotherapy in patients with metastatic castration-resistant prostate cancer that had progressed after docetaxel chemotherapy (CA184-043): a multicentre, randomised, double-blind, phase 3 trial. Lancet Oncol.

[2] Beer TM (2017). Randomized, double-blind, phase III trial of ipilimumab versus placebo in asymptomatic or minimally symptomatic patients with metastatic chemotherapy-naive castration-resistant prostate cancer. J Clin Oncol.

[3] Le DT (2015). PD-1 Blockade in tumors with mismatch-repair deficiency. N Engl J Med.

[4] Graff JN (2016). Early evidence of anti-PD-1 activity in enzalutamide-resistant prostate cancer. Oncotarget.

[5] Hansen A (2016). Pembrolizumab for patients with advanced prostate adenocarcinoma: Preliminary results from the KEYNOTE-028 study. Ann Oncol.

[6] Pritchard CC (2014). Complex MSH2 and MSH6 mutations in hypermutated microsatellite unstable advanced prostate cancer. Nat Commun.

[7] Nghiem B (2016). Mismatch repair enzyme expression in primary and castrate resistant prostate cancer. Asian J Urol.

[8] Nilbert M, Planck M, Fernebro E, Borg A, Johnson A (1999). Microsatellite instability is rare in rectal carcinomas and signifies hereditary cancer. Eur J Cancer.

[9] Davar D, Lin Y, Kirkwood JM (2015). Unfolding the mutational landscape of human melanoma. J Invest Dermatol.

[10] Alexandrov LB (2013). Signatures of mutational processes in human cancer. Nature.

[11] Van Allen EM (2015). Genomic correlates of response to CTLA-4 blockade in metastatic melanoma. Science.

[12] Robinson D (2015). Integrative clinical genomics of advanced prostate cancer. Cell.

[13] Salipante SJ, Scroggins SM, Hampel HL, Turner EH, Pritchard CC (2014). Microsatellite instability detection by next generation sequencing. Clin Chem.

[14] Hause RJ, Pritchard CC, Shendure J, Salipante SJ (2016). Classification and characterization of microsatellite instability across 18 cancer types. Nat Med.

[15] Mateo J (2015). DNA-Repair defects and olaparib in metastatic prostate cancer. N Engl J Med.

[16] Alexandrov LB (2016). Mutational signatures associated with tobacco smoking in human cancer. Science.

[17] Lawrence MS (2013). Mutational heterogeneity in cancer and the search for new cancer-associated genes. Nature.

[18] Kim J (2016). Somatic ERCC2 mutations are associated with a distinct genomic signature in urothelial tumors. Nat Genet.

[19] Lee DD, Seung HS (1999). Learning the parts of objects by non-negative matrix factorization. Nature.

[20] Newman AM (2015). Robust enumeration of cell subsets from tissue expression profiles. Nat Methods.

[21] Ramsay AG (2013). Immune checkpoint blockade immunotherapy to activate anti-tumour T-cell immunity. Br J Haematol.

[22] Cesano A (2015). nCounter® PanCancer Immune Profiling Panel (NanoString Technologies, Inc., Seattle, WA). J Immunother Cancer.

[23] Le DT (2017). Mismatch repair deficiency predicts response of solid tumors to PD-1 blockade. Science.

[24] Herbst RS (2014). Predictive correlates of response to the anti-PD-L1 antibody MPDL3280A in cancer patients. Nature.

[25] Bartley AN (2014). Template for reporting results of biomarker testing of specimens from patients with carcinoma of the colon and rectum. Arch Pathol Lab Med.

[26] Rimm DL (2017). A Prospective, Multi-institutional, pathologist-based assessment of 4 immunohistochemistry assays for PD-L1 expression in non-small cell lung cancer. JAMA Oncol.

[27] Roach C (2016). Development of a companion diagnostic PD-L1 immunohistochemistry assay for pembrolizumab therapy in non-small-cell lung cancer. Appl Immunohistochem Mol Morphol.

[28] Levenson RM, Fornari A, Loda M (2008). Multispectral imaging and pathology: seeing and doing more. Expert Opin Med Diagn.

[29] Van Allen EM (2014). Whole-exome sequencing and clinical interpretation of formalin-fixed, paraffin-embedded tumor samples to guide precision cancer medicine. Nat Med.

[30] DePristo MA (2011). A framework for variation discovery and genotyping using next-generation DNA sequencing data. Nat Genet.

[31] Cibulskis K (2013). Sensitive detection of somatic point mutations in impure and heterogeneous cancer samples. Nat Biotechnol.

[32] Garofalo A (2016). The impact of tumor profiling approaches and genomic data strategies for cancer precision medicine. Genome Med.

[33] Ramos AH (2015). Oncotator: cancer variant annotation tool. Hum Mutat.

[34] Lek M (2016). Analysis of protein-coding genetic variation in 60,706 humans. Nature.

[35] 1000 Genomes Project Consortium (2015). A global reference for human genetic variation. Nature.

[36] Sherry ST (2001). dbSNP: the NCBI database of genetic variation. Nucleic Acids Res.

[37] Forbes SA (2017). COSMIC: somatic cancer genetics at high-resolution. Nucleic Acids Res.

[38] Talevich E, Shain AH, Botton T, Bastian BC (2016). CNVkit: genome-wide copy number detection and visualization from targeted DNA sequencing. PLoS Comput Biol.

[39] Brunet JP, Tamayo P, Golub TR, Mesirov JP (2004). Metagenes and molecular pattern discovery using matrix factorization. Proc Natl Acad Sci U S A.

[40] Gehring JS, Fischer B, Lawrence M, Huber W (2015). SomaticSignatures: inferring mutational signatures from single-nucleotide variants. Bioinformatics.

[41] Gaujoux R, Seoighe C (2010). A flexible R package for nonnegative matrix factorization. BMC Bioinformatics.

[42] Johnson W (2007). Adjusting batch effects in microarray data using empirical Bayes methods. Biostatistics.

[43] Chakraborty S, Datta S, Datta S (2012). Surrogate variable analysis using partial least squares (SVA-PLS) in gene expression studies. Bioinformatics.

[44] Armenia J (2018). The long tail of oncogenic drivers in prostate cancer. Nat Genet.

[45] Youden WJ (1950). Index for rating diagnostic tests. Cancer.

