# Supplementary material for: Immunogenomic analyses associate immunological alterations with mismatch repair defects in prostate cancer
Source: J Clin Invest. 2018 Sep 4;128(10):4441–53. doi: 10.1172/JCI121924 (PMC6159966; doi:10.1172/JCI121924)
Supplement: Supplemental data [file jci-128-121924-s166.pdf]

Supplementary Figure 1

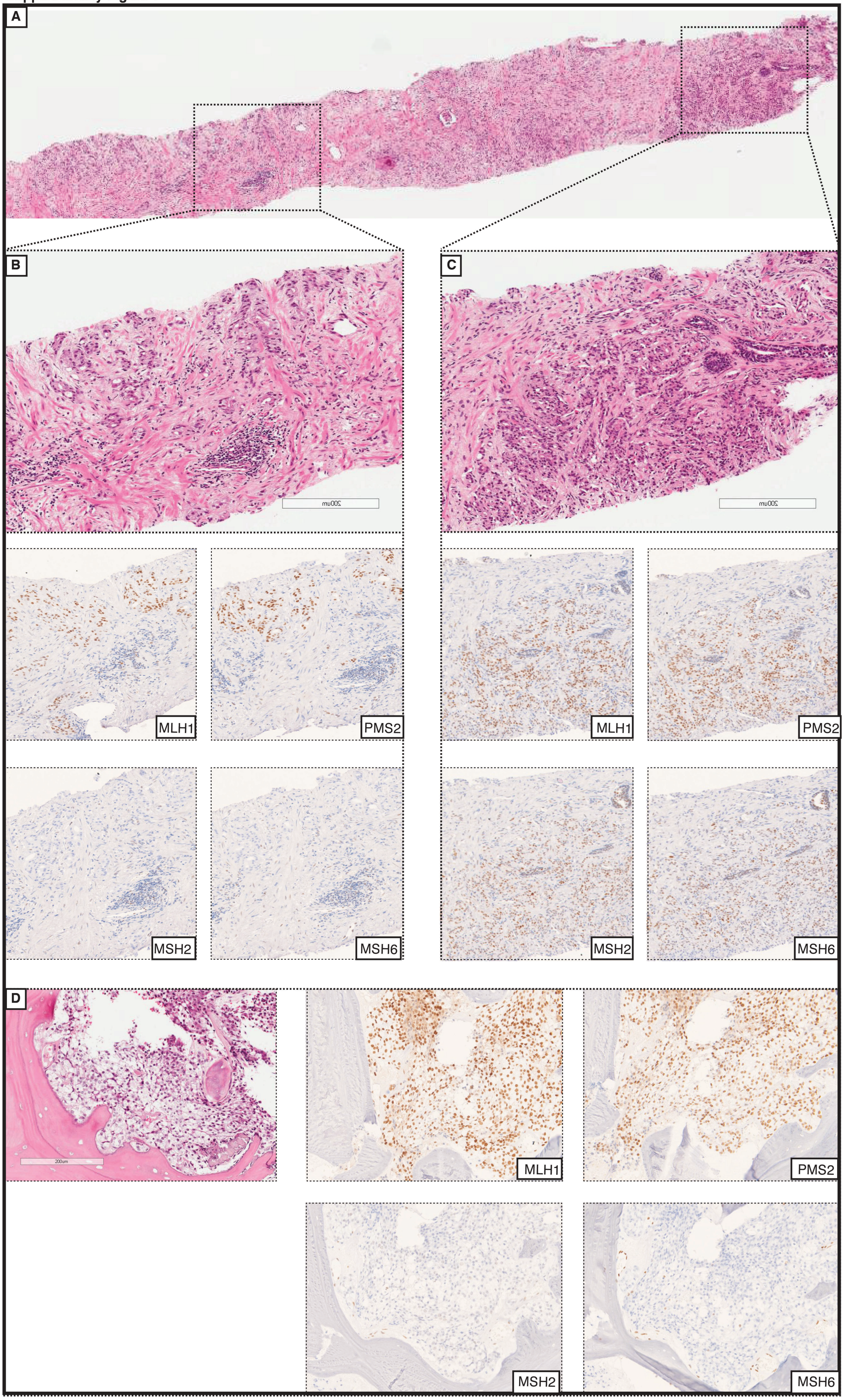

Supplementary Figure 2

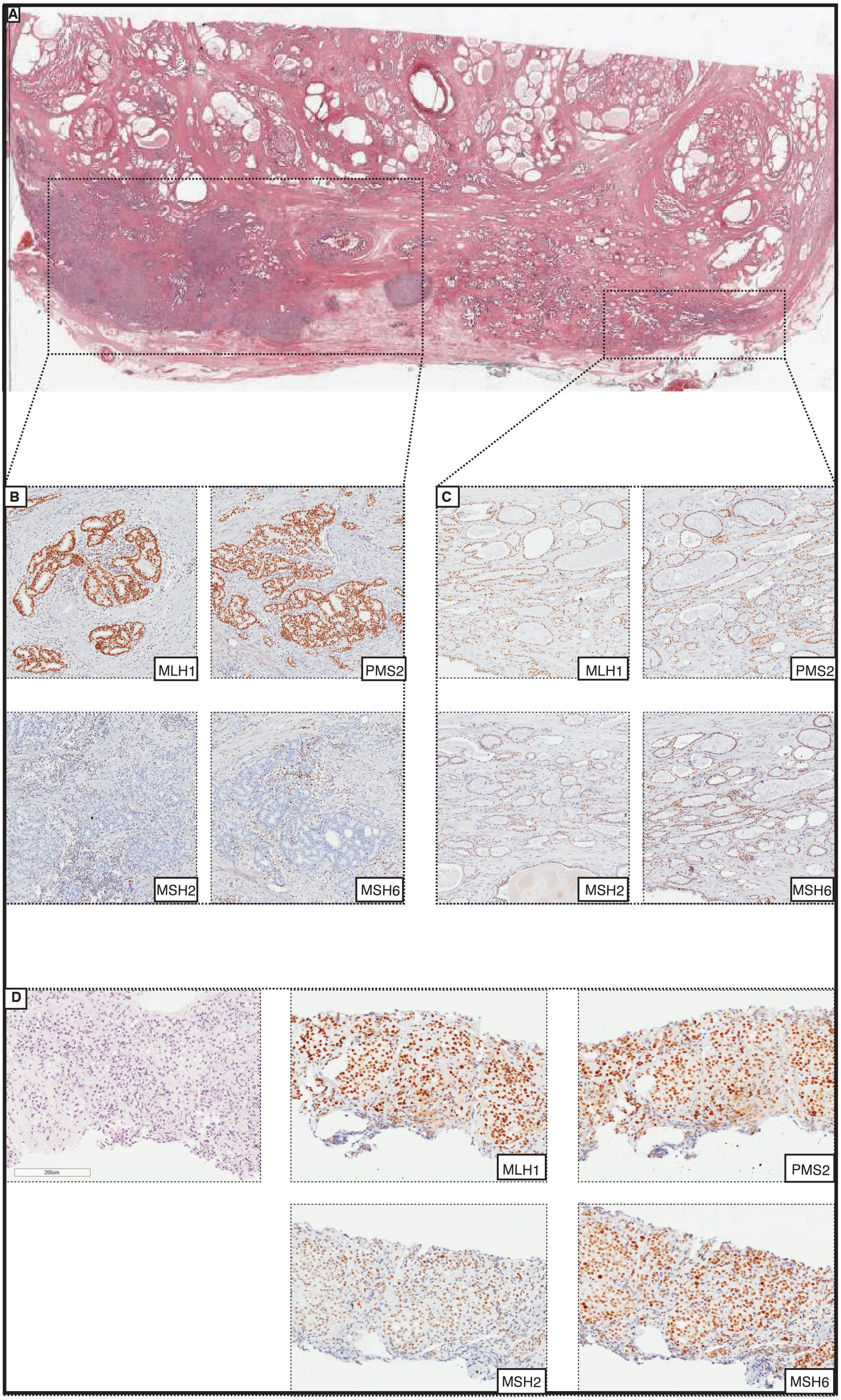

Supplementary Figures 1 and 2: Matched HSPC/mCRPC cases from two patients with dMMR heterogeneity in primary prostate cancer. Supplementary Figure 1. (**A**) Panel shows a needle biopsy specimen with two distinctly separate tumor foci (highlighted). These foci correspond to MSH2-MSH6 negative (2.5x magnification) (**B**) and positive (100x magnification) (**C**) tumors. (100x magnification) (**D**) A bone mCRPC specimen from this patient shows MSH2-MSH6 negative disease. (200x magnification) Supplementary Figure 2. (**A**) Panel shows a prostatectomy specimen with bilateral disease at scan magnification. (2.5x magnification) (**B**) A larger focus (highlighted) shows MSH2-MSH6 loss of expression while (100x magnification) (**C**) a smaller one (highlighted) shows retained staining. (100x magnification) (**D**)This patient's lymph node mCRPC sample shows retained staining for MSH2-MSH6 (200x magnification).

Supplementary Figure 3

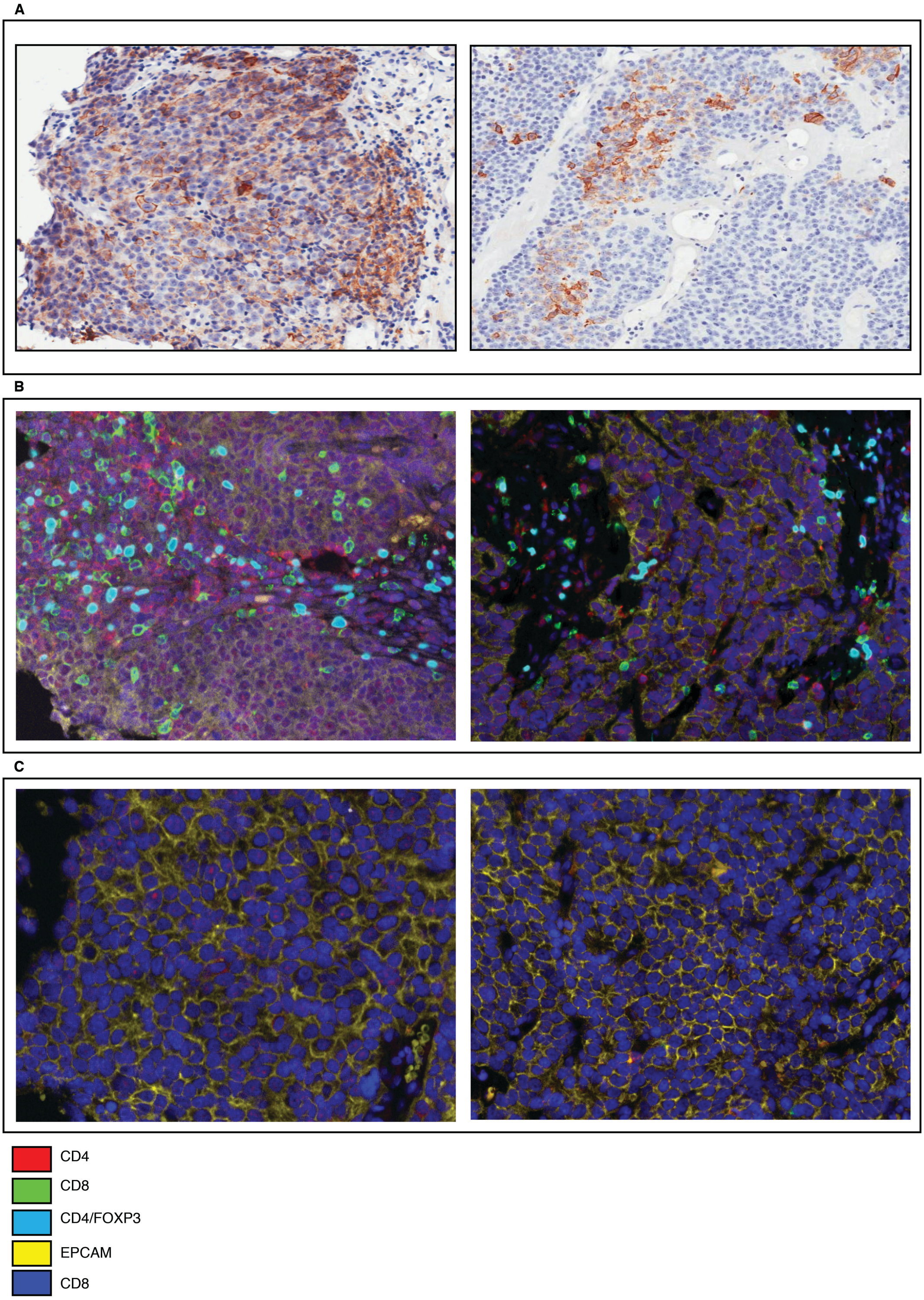

Supplementary Figure 3: Micrographs showing PD-L1 staining and T-cells infiltration from the RMH cohort (200x magnification). (**A**) Two examples of dMMR cases showing full membranous expression in most cells (left) and partial membranous expression in occasional cells (right) of PD-L1 by immunohistochemistry (200x magnification). (**B**) Multi-spectral, multi-color Immunofluorescence for T-cells infiltration in lymph nodes samples: dMMR tumor. (200x magnification) (**C**) Multi-spectral, multi-color Immunofluorescence for T-cells infiltration in lymph nodes samples: pMMR tumour (200x magnification).

Supplementary Figure 4

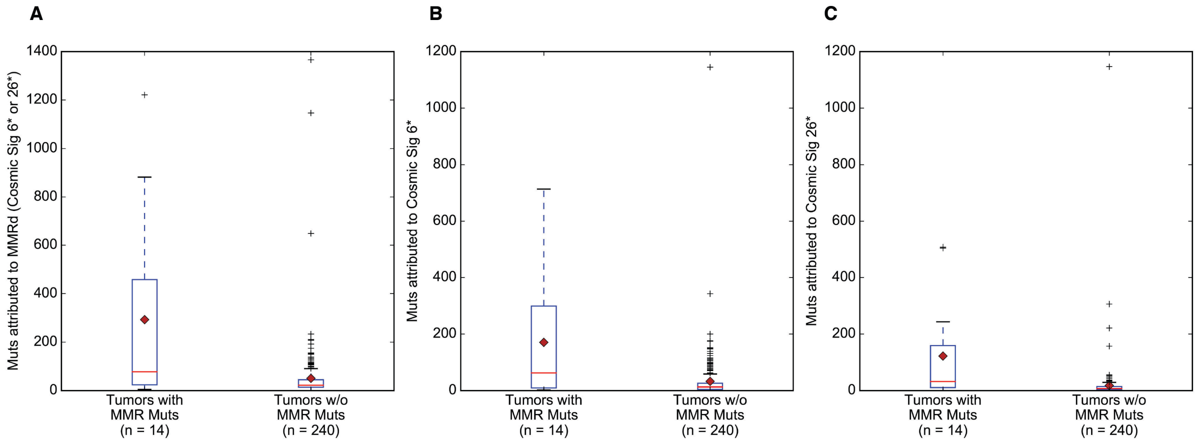

Supplementary Figure 4: Association between biallelic or nonsynonymous events in MMR genes and dMMR signatures in 254 tumors from PCF/SU2C dataset.

(**A**) Box and whiskers plot showing association between MMR mutations and number of mutations attributed to COSMIC signatures 6 or 26. (**B**) Box and whiskers plot showing association between MMR mutations and number of mutations attributed to COSMIC signatures 6. (**C**) Box and whiskers plot showing association between MMR mutations and number of mutations attributed to COSMIC signatures 26.

The box borders represent the 25th and 75th percentile values (interquartile range, IQR), with the median indicated by a line within the box. The whiskers represent values below the 25th percentile or above the 75th percentile within 1.5 x IQR from the respective boundaries. Additional dots represent outlier values outside this range.

Supplementary Figure 5

A

Transcriptome analyses of MMR6 mutational signature prostate cancers

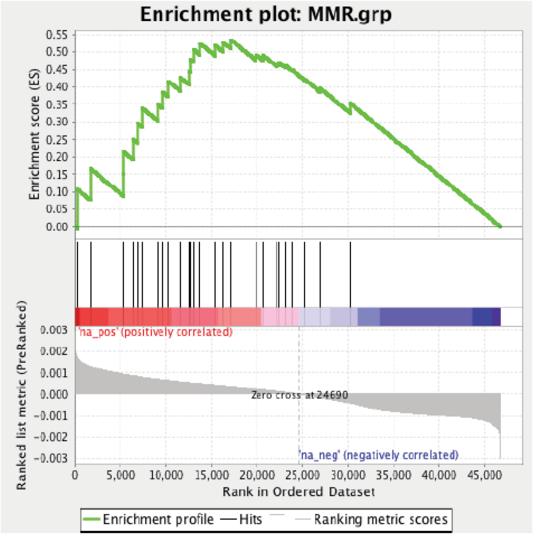

Gene	Coefficient
POLD1	1.7E-03
PMS1	1.3E-03
RFC3	9.3E-04
LIG1	9.3E-04
POLE	8.6E-04
RPA2	8.3E-04
PCNA	7.9E-04
RPA1	7.0E-04
RFC2	6.7E-04
MLH3	6.5E-04
RPA4	5.8E-04
POLD3	5.4E-04
EXO1	5.4E-04
SSBP1	5.3E-04
RFC4	5.0E-04
RFC5	4.4E-04
RPA3	4.0E-04
RFC1	3.7E-04
POLD2	2.5E-04
MSH6	2.1E-04
MSH5	1.4E-04
PMS2	1.2E-04
POLD4	8.7E-05
MLH1	4.9E-05
MSH3	-3.5E-05
MSH2	-1.5E-04
MSH4	-4.1E-04

GSEA:

Enrichment Score (ES): 0.53

Normalized (ES): 2.1

Nominal p value: $<1x10^{-4}$

B

Transcriptome analyses of MMR26 mutational signature prostate cancers

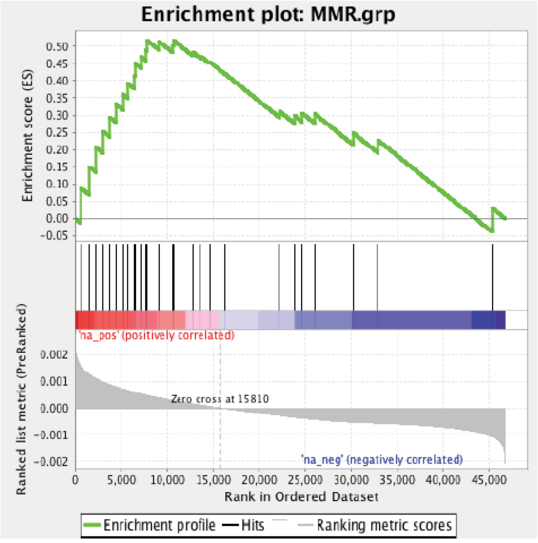

Gene	Coefficient
MSH4	1.6E-03
SSBP1	1.2E-03
PMS1	1.1E-03
RPA3	9.8E-04
RFC2	8.7E-04
POLD1	8.0E-04
PCNA	7.0E-04
RPA2	6.6E-04
RFC3	5.8E-04
RFC5	5.8E-04
POLD2	5.2E-04
MSH5	4.8E-04
RFC4	4.7E-04
MLH3	3.8E-04
LIG1	2.9E-04
RPA1	2.8E-04
EXO1	1.6E-04
POLD4	1.1E-04
POLE	5.9E-05
RFC1	-2.2E-05
POLD3	-2.8E-04
PMS2	-3.5E-04
MSH6	-3.8E-04
MSH3	-4.2E-04
MSH2	-5.3E-04
RPA4	-5.6E-04
MLH1	-1.1E-03

GSEA:

Enrichment Score (ES): 0.52

Normalized (ES): 2.0

Nominal p value: $<1x10^{-4}$

Supplementary Figure 5: Pathway enrichment analyses using transcriptome data in the PCF/SU2C dataset (n=168). (**A**) The bS1/nS1 signature was positively associated with a dMMR associated mRNA signature in APC transcriptomes. (**B**) bS2/nS2 signature was also positively associated with a dMMR associated mRNA signature in APC transcriptomes.

Supplementary Table 1
Genomic Alterations in MMR Genes (*MSH2, MSH6, MLH1, PMS2*)

Supplementary Table 2
Distribution of tumor sample sites for 176 CRPC tumors with transcriptome data.

Supplementary Table 3: 89 immune genes with expression significantly associated (FDR < 0.1) with dMMR signature activity in all samples.

Supplementary Table 4: 55 immune genes with expression significantly associated (FDR < 0.1) with dMMR signature activity in bone metastases.

Supplementary Table 5: 36 immune genes with expression significantly associated (FDR < 0.1) with dMMR signature activity in lymph node metastases.

Supplementary Table 6: Association (p-value) of all 762 immune genes with dMMR signature activity in all tumors, bone metastases only, and lymph node metastases only.
